# Content of Manganese and Molybdenum in Selected Tissues and Organs of Mallards (*Anas platyrhynchos* L.) in Relation to Their Sex and Harvest Location

**DOI:** 10.3390/ani16213325

**Published:** 2026-10-22

**Authors:** Elżbieta Bombik, Antoni Bombik, Katarzyna Pietrzkiewicz

**Affiliations:** Faculty of Agricultural Sciences, University of Siedlce, Prusa 14, 08-110 Siedlce, Poland; antoni.bombik@uws.edu.pl (A.B.); pietrzkiewicz.ka@gmail.com (K.P.)

**Keywords:** wild mallard, breast muscle, leg muscle, liver, manganese, molybdenum

## Abstract

The aim of this study was to analyze the manganese and molybdenum content in selected tissues of mallard ducks (*Anas platyrhynchos* L.) depending on their location of collection and gender. The study was conducted on material obtained from mallard ducks from two study areas: the Siedlce hunting district and the Leszno hunting district. The research material consisted of breast muscles, leg muscles, and liver obtained from 28 mallards. The samples were frozen and stored at −20 °C. The material was analysed for manganese and molybdenum using inductively coupled argon plasma atomic emission spectroscopy (ICP OES), with a Perkin Elmer Optima 2000 DV spectrometer (Shelton, WA, USA). Manganese levels were significantly higher in mallards harvested in the Siedlce hunting district than in those from the Leszno hunting district. The pattern was reversed for molybdenum, which was present in higher concentrations in the tissues of mallards from the Leszno hunting district, although these were not statistically significant differences. Among the muscles and organs tested, the highest levels of manganese and molybdenum were shown in the liver. The level of molybdenum in the liver was significantly higher in male mallards than in females. An effect of the birds’ origin on manganese content and of sex on molybdenum content in mallard tissues was observed.

## Simple Summary

Manganese and molybdenum are heavy metals. These trace elements are essential for human, animal, and plant life in small amounts, but can be toxic in excess. Changes in the habitat of wild mallard ducks, and therefore their diet, can lead to differences in manganese and molybdenum content in their tissues and organs. This study analyzed manganese and molybdenum content in selected tissues and organs of wild mallard ducks (*Anas platyrhynchos* L.) depending on their sex and collection location.

## 1. Introduction

Manganese (Mn), unlike other heavy metals, is in certain quantities essential to the normal functioning of higher organisms [1]. Mn supports the immune system, regulates blood sugar levels, and takes part in energy production and cell reproduction. It is an activator of numerous enzymes, including pyruvate carboxylase, liver arginase, which catalyses urea synthesis, and alkaline phosphatase (ALP), which promotes calcium metabolism [2,3]. It is involved in metabolic transformations of lipids and carbohydrates, protein biosynthesis, and cartilage formation. Mn is absorbed in the small intestine and excreted mainly in the urine [4,5]. Deficiencies of this element, in both humans and animals, lead to inhibition of growth of young organisms, including disorders of skeletal development and bone tissue maturation, as well as to reduced fertility [4,6]. An excess of manganese exerts toxic effects on the nervous system and circulatory system. Disease symptoms caused by excess manganese in the body manifest as fatigue, hyperactivity, increased blood pressure, clotting and fibrinolysis disorders, and unfavourable morphological and functional changes in the heart muscle [7]. According to nutritional standards for the Polish population, no daily manganese requirement has been determined, but only adequate intake (AI). The need for manganese depends on sex and age. Women and men at the age of 31–50 require 1.8 mg and 2.3 mg a day, respectively, while pregnant women require 2 mg per day [8].

Molybdenum (Mo) is mainly present in the bones, kidneys, spleen, and liver [4,9]. The amount of Mo present in the human body is about 10 times that of chromium (9.3 mg vs. 1.8 mg in a person with a body weight of 70 kg) and similar to the level of manganese (12 mg) [10]. It is excreted mainly in the urine [11]. Its level of absorption depends on multiple factors, including the chemical form of Mo and the species of animal, ranging from 75% to 95% in animals and from 28% to 77% in humans [9]. Molybdenum is a component of numerous enzymes, including xanthine oxidase, aldehyde oxidase, and sulfite oxidase [12]. It is involved in purine metabolism and interacts with cytochrome c. When present in large amounts in the body, Mo exerts toxic effects and can lead to the occurrence of gout [11]. An excessive amount of this element in the diet can cause disturbances in Cu absorption by the body.

In Poland, about 10% of soils are poor in manganese and molybdenum deficiencies occur on about 20% of soil surfaces [13]. Manganese is an element commonly found in Poland’s aquatic environment; it occurs naturally in soil and rocks, from which it easily penetrates into water. Molybdenum is a rare metal, and its natural concentration in Poland’s aquatic environment is minimal [14]. The literature lacks information on manganese and molybdenum levels in the tissues of mallard ducks in relation to their habitat. The aim of the study was to analyse the content of manganese and molybdenum in selected tissues and organs of wild mallards (*Anas platyrhynchos* L.) in relation to their sex and harvest location.

## 2. Materials and Methods

### 2.1. Animals and Sampling

The material for the study consisted of breast muscles, leg muscles, and livers of mallards (*Anas platyrhynchos* L.) harvested in two study areas, i.e., in the Siedlce and Leszno hunting districts. The two areas had different environmental resources and different levels of contamination with heavy metals [15]. Cereals (wheat, barley, and maize) dominate the crop structure in both the Siedlce hunting district (Masovian Voivodeship) and the Leszno hunting district (Greater Poland Voivodeship). Cereal grains made up a significant portion of the mallards’ diet. Large farms engaged in intensive crop production using significant amounts of mineral fertilizers—resulting in higher environmental exposure—predominate in the Greater Poland Voivodeship. Farms in the Masovian Voivodeship are characterized by smaller surface areas and more balanced farming practices, resulting in a lower environmental impact. In both the Siedlce and Leszno hunting districts, duck hunting took place at fish ponds. These were primarily standing waters, including naturally formed field ponds and commercial fish-rearing ponds. Six male and six female mallards were obtained from the Siedlce hunting district, and eight males and eight females from the Leszno hunting district. Mallard hunting was limited to the first two months of the hunting season for this species—specifically, from 15 August to 15 October—prior to the onset of the birds’ migration. The age of the ducks was assessed based on the hardness of their bills. The majority were young (approximately one year old) and had bills with a more pliable texture. The animals were obtained during legal hunts conducted by licensed hunters, in accordance with the Polish Hunting Law [16] and applicable permits issued by the relevant leaseholders or managers of hunting grounds. Muscle samples were collected post-mortem with the consent of the persons authorized to dispose of the obtained animals. No procedures were performed on live animals for the purposes of this study; therefore, in accordance with the Polish Act of 15 January 2015 on the protection of animals used for scientific or educational purposes [17], the consent of the Local Ethical Committee for Animal Experiments was not required.

### 2.2. Laboratory Analyses

The material was analysed for manganese and molybdenum using inductively coupled argon plasma atomic emission spectroscopy (ICP OES), with a Perkin Elmer Optima 2000 DV spectrometer (Shelton, WA, USA). After thawing, the material was homogenized in an agate mortar. Weighted samples of about 1 g were transferred to quartz pressure vessels, to which 5.0 mL of 65% HNO_3_ (Suprapur™, Merck KGaA, Darmstadt, Germany) and 1 mL 30% H_2_O_2_ (Suprapur™, Merck KGaA, Darmstadt, Germany) were added. Then the samples were digested in an Anton Paar microwave system (Anton Paar GmbH, Graz, Austria). Tightly sealed vessels were placed in the mineralizer, which was equipped with a constant temperature and pressure control system. Digestion was carried out under the following conditions: 0–5 min—linear power gradient 100–600 W; 6–10 min—600 W (const.); 11–20 min—1000 W or less after reaching the pressure limit (75 MPa) or temperature limit (300 °C); 21–35 min—cooling to ambient temperature. The cooled and degassed (CO_2_, NO_2_) digest was made up to 10 mL in class A volumetric flasks (BRAND, Wertheim, Germany), and radiation emissions were measured in the solutions. The analytical wavelengths and detection limits were 257.610 nm and DL 0.1 μg·L^−1^ for Mn, and 202.030 nm and DL 0.5 μg·L^−1^ for Mo. The longer axial optical path (along the plasma) was chosen for the microelements manganese and molybdenum.

### 2.3. Statistical Methods

For each trait in each group, i.e., for hunting districts and mallard sex, basic descriptive statistical measures were determined, i.e., arithmetic mean ($\bar{x}$ range of variation (x_min._–x_max._), standard deviation (s), and coefficient of variation (V%).

In addition, two-way nonorthogonal analysis of variance (Fisher–Snedecor F-test) with interaction was performed according to the following mathematical model:

y_ijl_ = m + a_i_ + b_j_ + ab_ij_ + e_ijl_,

where

y_ijl_—value of trait for ith hunting district (a = 2), jth sex (b = 2), and lth replicate (measurement);

m—population mean;

a_i_, b_j_—main effects of factors, i.e., hunting district and sex;

ab_ij_—effect of interaction of hunting district and sex;

e_ijl_—sampling error.

Significant effects were compared by Tukey’s test for a significance value of 0.05. Statistical analysis of the results was carried out using Statistica 13.0 software [18].

## 3. Results

The results for manganese content in selected tissues and organs of mallard ducks (*Anas platyrhynchos* L.) are presented in Table 1 and Table 2.

Analysis of the average manganese concentration in the breast muscles of mallards was 1.5 times as high in females (0.4014 mg·kg^−1^ WW) as in males (0.2489 mg·kg^−1^ WW), although the differences were not statistically significant (Table 1). In the breast muscle of mallards harvested in the Siedlce hunting district, manganese content (0.6654 mg·kg^−1^ WW) was significantly higher (more than tenfold) than in specimens from the Leszno hunting district (0.0647 mg·kg^−1^ WW). Greater variation was shown for the manganese concentration in the breast muscle of birds harvested in the Leszno hunting district (71.90%) than in birds from the Siedlce hunting district (58.26%). In the Siedlce and Leszno hunting districts, the average manganese concentration in the breast muscle was higher in female mallards than in males, although the differences were not statistically significant (Table 2).

The average manganese content in the leg muscles of mallards was higher in females (0.4158 mg·kg^−1^ WW) than in males (0.3771 mg·kg^−1^ WW), but these were not statistically significant differences (Table 1). Mallards harvested in the Siedlce hunting district had significantly higher (nearly fivefold) manganese concentration in the leg muscles (0.6934 mg·kg^−1^ WW) than specimens from the Leszno hunting district (0.1737 mg·kg^−1^ WW). The coefficient of variation of the manganese level in the leg muscles of mallards was highly varied, from 65.46% in the Siedlce hunting district to 200.15% in the Leszno hunting district. In the Siedlce hunting district, the average manganese level in the leg muscles was higher in female mallards (0.8437 mg·kg^−1^ WW) than in males (0.5431 mg·kg^−1^ WW) (Table 2), while the pattern was reversed in the Leszno hunting district. In this hunting district, the average manganese level in this tissue in males (0.2526 mg·kg^−1^ WW) was 2.5 times the level noted in females (0.0948 mg·kg^−1^ WW). In both hunting districts, the differences in the level of manganese in the muscles for sex were statistically non-significant.

The average manganese level in the liver of mallards was higher in males (3.5110 mg·kg^−1^ WW) than in females (3.0267 mg·kg^−1^ WW), although these were not statistically significant differences (Table 1). The Mn content in the liver was significantly higher—nearly twofold—in mallards from the Siedlce hunting district (4.3193 mg·kg^−1^ WW) than in birds from the Leszno hunting district (2.4809 mg·kg^−1^ WW). The coefficient of variation of manganese content in the liver was 21.97% for the Leszno hunting district and 33.55% for the Siedlce hunting district. In the Siedlce hunting district, the manganese level in the liver was higher in male mallards than in females (Table 2), while the pattern was reversed in the Leszno hunting district; the average value for this trait was higher in females than in males. In both hunting districts, these differences were statistically non-significant.

The results for molybdenum in the tissues and organs of mallards (*Anas platyrhynchos* L.) are presented in Table 3 and Table 4.

The analysis showed no significant differences in the molybdenum level in the breast muscle between male (0.0093 mg·kg^−1^ WW) and female (0.0092 mg·kg^−1^ WW) mallards (Table 3). The average concentration was higher in the breast muscle of mallards harvested in the Leszno hunting district (0.0095 mg·kg^−1^ WW) than in that of specimens from the Siedlce hunting district (0.0089 mg·kg^−1^ WW). In the Siedlce hunting district, the average molybdenum level in the breast muscle was higher in female mallards than in males (Table 4), while in the Leszno hunting district, it was higher in males than in females. The differences were statistically non-significant in both hunting districts.

There were no significant differences in the molybdenum level in the leg muscles between female (0.0090 mg·kg^−1^ WW) and male (0.0088 mg·kg^−1^ WW) mallards (Table 3). The average molybdenum concentration was higher in the leg muscles of mallards harvested in the Leszno hunting district (0.0091 mg·kg^−1^ WW) than in those of birds from the Siedlce hunting district (0.0086 mg·kg^−1^ WW), but these differences were statistically non-significant. In the Siedlce hunting district, the mean molybdenum level was higher in male mallards than in females (Table 4), while the pattern was reversed in the Leszno hunting district. These differences were statistically non-significant in both hunting districts.

The average molybdenum content in the liver was significantly higher in male mallards (0.0292 mg·kg^−1^ WW) than in females (0.0231 mg·kg^−1^ WW) (Table 3). The variation in the molybdenum concentration in the liver was more than twice as high for males (19.13%) as for females (8.67%). The average molybdenum level in the liver of birds harvested in the Leszno hunting district (0.0264 mg·kg^−1^ WW) was higher than in birds from the Siedlce hunting district (0.0258 mg·kg^−1^ WW), but these differences were not statistically significant. In both hunting districts, the average molybdenum level in the liver of mallards was higher in males than in females, although these differences were not statistically significant (Table 4).

## 4. Discussion

Kalisińska et al. [19] showed age-dependent differences in manganese levels in the liver of mallards harvested in the vicinity of Szczecin. Adult birds showed higher values (average content 5.99 mg·kg^−1^ WW) than juveniles (4.51 mg·kg^−1^ WW), and these values were higher than the average manganese content in the liver of wild mallards in the present study, which was 2.4809 mg·kg^−1^ WW in the Leszno hunting district and 4.3193 mg·kg^−1^ WW in the Siedlce hunting district. The lower average manganese content in the liver of mallard ducks obtained in our study could be due to the fact that most of them were young individuals. The average manganese content in the breast muscle of mallards harvested in the Słońsk Nature Reserve was 1.33 mg·kg^−1^ WW for adults and 1.09 mg·kg^−1^ WW for juveniles; this was twice as high as in adult and juvenile mallards from the vicinity of Szczecin (0.60 mg·kg^−1^ WW and 0.55 mg·kg^−1^ WW, respectively). According to the authors, the differences may be linked to the specific character of the habitat. These are field ponds located in the vicinity of Szczecin, which are subject to contamination with manganese from agriculture and diesel fuel combustion. In this study, the average manganese content in the breast muscles of mallards was lower and ranged from 0.0647 mg·kg^−1^ WW in the Leszno hunting district to 0.6654 mg·kg^−1^ WW in the Siedlce hunting district. The location where the ducks were obtained may have played a role. The ponds where mallards were hunted in the Siedlce hunting district were surrounded by agricultural fields; hence the exposure to manganese pollution could have been greater. Sun et al. [1], in an analysis of the effect of dietary supplementation with manganese hydroxychloride (MHC) on manganese content in broiler tissues at 42 days of rearing, reported much lower average content of Mn in the breast muscle than was shown in the present study. The authors found that manganese content in the breast muscle ranged from 0.37 mg kg^−1^ DW (0.0814 mg·kg^−1^ WW) for broilers in the control group to 0.42 mg kg^−1^ DW (0.0924 mg·kg^−1^ WW) for birds receiving 100 mg·kg^−1^ Mn in the form of MnSO4. The average manganese content in the muscles of common kestrel (*Falco tinnunculus*) from the Canary Islands (Spain), amounting to 0.760 mg·kg^−1^ DW (0.1672 mg·kg^−1^ WW) [20], was similar to the results of the present study and the findings of other authors: 0.883–2.07 mg·kg^−1^ DW (0.1942–0.4554 mg·kg^−1^ WW) [21] and 0.55 mg·kg^−1^ DW (0.121 mg·kg^−1^ WW) [22]. Rodríguez-Álvarez et al. [20] compared the average content of metals in the liver and muscles of common kestrel and found the highest levels of most trace elements in the liver, including Mn and Mo. This is supported by the present study. The average manganese content in the liver of common kestrels was 2.698 mg·kg^−1^ DW (0.5936 mg·kg^−1^ WW), which was lower than the values obtained in the present study. Lower Mn levels in the tissues of the common kestrel may result from the different diet of this bird of prey compared to the mallard, a bird associated with the aquatic environment. Bojar and Bojar [23] found, consistent with the results of the present study, that the average manganese level in the liver of mallards was higher than in the breast muscle. The average manganese level in the liver of the birds ranged from 0.88 mg·kg^−1^ WW (individuals harvested in the vicinity of Mosty) to 2.04 mg·kg^−1^ WW (vicinity of Częstoborowice). The corresponding values in the breast muscle ranged from 0.21 mg·kg^−1^ WW (mallards harvested in the vicinity of Przytoczno) to 0.29 mg·kg^−1^ WW (vicinity of Częstoborowice). Dżugan et al. [24], in a study of pheasants harvested in the vicinity of Rzeszów, showed average manganese levels of 0.162 mg·kg^−1^ WW in the breast muscle and 2.839 mg·kg^−1^ WW in the liver. In a study by Sujak et al. [25], the average manganese concentration in the liver of mallards wintering in south-eastern Poland was higher than in the present study, amounting to 17.89 mg·kg^−1^ DW. Kim and Oh [26] found that the average manganese content in the liver was influenced by the species of bird. They showed that its level in the greater white-fronted goose (28.1 mg·kg^−1^ DW) was four times the value obtained for mallards (6.92 mg·kg^−1^ DW). Majewska et al. [27] demonstrated differences in the mean manganese concentration in the liver of various poultry species, with the highest value obtained in turkeys (3.69 mg·kg^−1^ WW), followed by chicken broilers (2.48 mg·kg^−1^ WW), while the lowest value was shown for ostriches (1.83 mg·kg^−1^ WW). Sun et al. [1], in their analysis of the effect of dietary supplementation with manganese hydroxychloride (MHC) on the manganese content in chicken broilers at 42 days of rearing, showed that the manganese level in the liver of these birds ranged from 9.16 mg·kg^−1^ DW (2.0152 mg·kg^−1^ WW) for broilers in the control group to 11.66 mg·kg^−1^ DW (2.5652 mg·kg^−1^ WW) for birds receiving 100 mg·kg^−1^ Mn as MHC. Durkalec et al. [28] showed a difference in manganese content in the liver between golden eagles and white-tailed eagles, in which the estimated means were 22.38 and 13.90 mg·kg^−1^ DW, respectively (4.9236 and 3.058 mg·kg^−1^ WW). The Mn levels obtained in that study correspond to the results of the present study and those reported by other authors for common buzzards—9.18 mg·kg^−1^ DW (2.0196 mg·kg^−1^ WW)—and common kestrels—14.3 mg·kg^−1^ DW (3.146 mg·kg^−1^ WW)—in Korea [29] and for raptors in Poland [30]. The average manganese content in the liver of various wild birds in Iran ranged from 10 to 16 mg·kg^−1^ DW (2.2 to 3.52 mg·kg^−1^ WW) [31].

The present study did not show statistically significant differences between the average molybdenum level in the breast muscle of mallards harvested in the Leszno hunting district (0.0095 mg·kg^−1^ WW) and the Siedlce hunting district (0.0089 mg·kg^−1^ WW). The average molybdenum content in these muscles was similar in males (0.0093 mg·kg^−1^ WW) and females (0.0092 mg·kg^−1^ WW). The molybdenum level in the breast muscle of mallards ranged from 0.0072 mg·kg^−1^ WW to 0.0117 mg·kg^−1^ WW. Also in the case of leg muscles of mallards, no significant differences were shown in the molybdenum level between birds from the Leszno hunting district (0.0091 mg·kg^−1^ WW) and Siedlce hunting district (0.0086 mg·kg^−1^ WW) or between females (0.0090 mg·kg^−1^ WW) and males (0.0088 mg·kg^−1^ WW). The level of this element in the leg muscles of mallards ranged from 0.0069 mg·kg^−1^ WW to 0.0116 mg·kg^−1^ WW. Rodríguez-Álvarez et al. [20], in their analysis of the muscles (breast and coracobrachialis muscle) of kestrels from the Canary Islands, reported a similar average Mo level—0.013 mg·kg^−1^ DW (0.0029 mg·kg^−1^ WW)—to that obtained in the present study.

Among all tissues and organs tested in mallards, the liver had the highest molybdenum content. The average molybdenum content in this organ was similar in mallards harvested in the Leszno hunting district (0.0264 mg·kg^−1^ WW) and the Siedlce hunting district (0.0258 mg·kg^−1^ WW). The average content of molybdenum in the liver was significantly higher in male mallards (0.0292 mg·kg^−1^ WW) than in females (0.0231 mg·kg^−1^ WW). The average Mo level in the liver of kestrels from the Canary Islands was 0.352 mg·kg^−1^ DW (0.0774 mg·kg^−1^ WW) [20], which was higher than the values obtained in the present study. These differences could have been caused by environmental conditions or the different diets of these species.

## 5. Conclusions

In summary, the study results revealed an effect of sex on molybdenum content and harvesting location on manganese content in mallard tissues. The location where the mallards were harvested may have influenced the statistically significantly higher mean manganese levels found in their tissues in the Siedlce hunting district compared to the Leszno district. The ponds where mallards were hunted in the Siedlce hunting district were surrounded by agricultural fields, which could have influenced the degree of eutrophication of water bodies and the diversification of the food composition consumed by the birds in this area. The lack of significant differences in mean molybdenum levels across the examined tissues and organs of this wild bird may be attributed to the fact that its natural concentration in Poland’s aquatic environment is negligible. Among the muscles and organs tested, the highest levels of manganese and molybdenum were shown in the liver, which may indicate the accumulation of the heavy metals under study in this organ. The level of molybdenum in the liver was significantly higher in male mallards than in females.

## Figures and Tables

**Table 1:** Mean manganese content in selected tissues and organs of mallard ducks (*Anas platyrhynchos* L.) (mg·kg^−1^ WW).

Tissue/Organ	Basic Statistics	Sex		LSD_0.05_	Hunting District		LSD_0.05_
		Females (*n* = 14)	Males (*n* = 14)		Siedlce (*n* = 12)	Leszno (*n* = 16)	
Breast muscle	$\bar{x}$ min.–max. s V%	0.4014 a 0.0402–1.3797 0.4301 107.15	0.2489 a 0.0054–1.0758 0.3324 136.87	n. s.	0.6654 b 0.0222–1.3797 0.3876 58.26	0.0647 a 0.0054–0.1564 0.0465 71.90	0.2010
Leg muscle	$\bar{x}$ min.–max. s V%	0.4158 a 0.0113–1.8360 0.5046 121.35	0.3771 a 0.0069–1.4793 0.4378 116.10	n. s.	0.6934 b 0.1378–1.8360 0.4539 65.46	0.1737 a 0.0069–1.4793 0.3477 200.15	0.3240
Liver	$\bar{x}$ min.–max. s V%	3.0267 a 1.5123–7.2938 1.3565 44.81	3.5110 a 1.5354–5.6121 1.3557 38.61	n. s.	4.3193 b 1.5123–7.2938 1.4493 33.55	2.4809 a 1.5354–3.5575 0.5450 21.97	0.8220

**Legend:** $\bar{x}$—arithmetic mean; min., max.—extreme values; s—standard deviation; V%—coefficient of variation; LSD_0.05_—least significant difference (*p* ≤ 0.05); a, b—means marked with different letters for sex and hunting district are significantly different (*p* ≤ 0.05). n. s.—no significance.

**Table 2:** Mean manganese content in selected tissues and organs of mallard ducks (*Anas platyrhynchos* L.) (mg·kg^−1^ WW) within hunting districts.

Tissue/Organ	Basic Statistics	Hunting District				LSD_0.05_
		Siedlce		Leszno		
		Females (*n* = 6)	Males (*n* = 6)	Females (*n* = 8)	Males (*n* = 8)	
Breast muscle	$\bar{x}$ min.–max. s V%	0.8165 a 0.2901–1.3797 0.3590 43.97	0.5143 a 0.0222–1.0758 0.3549 69.01	0.0900 a 0.0402–0.1333 0.0284 31.57	0.0393 a 0.0054–0.1564 0.0472 120.11	n. s.
Leg muscle	$\bar{x}$ min.–max. s V%	0.8437 a 0.3003–1.8360 0.5138 60.90	0.5431 a 0.1378–1.0606 0.3207 59.05	0.0948 a 0.0113–0.2882 0.0849 89.48	0.2526 a 0.0069–1.4793 0.4714 186.59	n. s.
Liver	$\bar{x}$ min.–max. s V%	3.7440 a 1.5122–7.2938 1.7523 46.80	4.8946 a 3.5516–5.6121 0.6845 13.98	2.4886 a 1.8051–3.5575 0.4917 19.76	2.4732 a 1.5354–3.4240 0.5934 24.00	n. s.

Legend as for Table 1.

**Table 3:** Mean molybdenum content in selected tissues and organs of mallard ducks (*Anas platyrhynchos* L.) (mg·kg^−1^ WW).

Tissue/Organ	Basic Statistics	Sex		LSD_0.05_	Hunting District		LSD_0.05_
		Females (*n* = 14)	Males (*n* = 14)		Siedlce (*n* = 12)	Leszno (*n* = 16)	
Breast muscle	$\bar{x}$ min.–max. s V%	0.0092 a 0.0076–0.0105 0.0008 9.07	0.0093 a 0.0072–0.0117 0.0011 12.18	n. s.	0.0089 a 0.0076–0.0105 0.0009 9.84	0.0095 a 0.0072–0.0117 0.0010 10.58	n. s.
Leg muscle	$\bar{x}$ min.–max. s V%	0.0090 a 0.0069–0.0116 0.0013 13.96	0.0088 a 0.0074–0.0113 0.0011 12.11	n. s.	0.0086 a 0.0069–0.0101 0.0011 12.44	0.0091 a 0.0074–0.0116 0.0012 13.20	n. s.
Liver	$\bar{x}$ min.–max. s V%	0.0231 a 0.0193–0.0267 0.0020 8.67	0.0292 b 0.0225–0.0411 0.0056 19.13	0.0030	0.0258 a 0.0200–0.0411 0.0056 21.74	0.0264 a 0.0192–0.0363 0.0048 18.18	n. s.

Legend as for Table 1.

**Table 4:** Mean molybdenum content in selected tissues and organs of mallard ducks (*Anas platyrhynchos* L.) (mg·kg^−1^ WW) within hunting districts.

Tissue/Organ	Basic Statistics	Hunting District				LSD_0.05_
		Siedlce		Leszno		
		Females (*n* = 6)	Males (*n* = 6)	Females (*n* = 8)	Males (*n* = 8)	
Breast muscle	$\bar{x}$ min.–max. s V%	0.0089 a 0.0076–0.0105 0.0010 11.46	0.0088 a 0.0079–0.0097 0.0007 7.84	0.0094 a 0.0082–0.0103 0.0006 6.28	0.0096 a 0.0072–0.0117 0.0013 13.37	n. s.
Leg muscle	$\bar{x}$ min.–max. s V%	0.0086 a 0.0069–0.0101 0.0012 14.17	0.0087 a 0.0078–0.0100 0.0009 10.40	0.0093 a 0.0080–0.0116 0.0012 12.66	0.0088 a 0.0074–0.0113 0.0012 13.19	n. s.
Liver	$\bar{x}$ min.–max. s V%	0.0229 a 0.0200–0.0250 0.0017 7.45	0.0288 a 0.0238–0.0411 0.0065 22.69	0.0233 a 0.0193–0.0267 0.0022 9.35	0.0295 a 0.0225–0.0363 0.0047 16.03	n. s.

Legend as for Table 1.

## Data Availability

The original contributions presented in this study are included in the article. Further inquiries can be directed to the corresponding author.

## References

[1] Sun Y., Geng S., Yuan T., Liu Y., Zhang Y., Di Y., Li J., Zhang L. (2021). Effects of Manganese Hydroxychloride on Growth Performance, Antioxidant Capacity, Tibia Parameters and Manganese Deposition of Broilers. Animals.

[2] Dismukes G.C. (1996). Manganese enzymes with binuclear active sites. Chem. Rev..

[3] Świątkiewicz S. (2005). Effect of the level and form of zinc and manganese in feed for layer hens on eggshell and bone quality and excretion of Zn and Mn. Rocz. Nauk. Zoot. Rozpr. Habilitacyjne.

[4] Jamroz D. (2013). Żywienie Zwierząt i Paszoznawstwo.

[5] Spears J.W. (2019). Boron, chromium, manganese, and nickel in agricultural animal production. Biol. Trace Elem. Res..

[6] Wang Z., Wang L., Wang Z., Wang J., Liu R. (2013). Effects of manganese deficiency on serum hormones and biochemical markers of bone metabolism in chicks. J. Bone Miner. Metab..

[7] Gać P., Waliszewska M., Zawadzki M., Poręba R. (2009). Narażenie na mangan. Bezp. Pr. Nauk Prakt..

[8] Wojtasik A., Woźniak A., Stoś K., Jarosz M., Jarosz M., Rychlik E., Stoś K., Charzewska J. (2020). Składniki Mineralne. W: Normy Żywienia dla Populacji Polskiej—Nowelizacja. Red. Nauk.

[9] Vyskočil A., Viau C. (1997). Assessment of molybdenum toxicity in humans. J. Appl. Toxicol..

[10] Firth G., Blower J.E., Bartnicka J.J., Mishra A., Michaels A.M., Rigby A., Darwesh A., Al-Salemee F., Blower P.J. (2022). Non-invasive radionuclide imaging of trace metal trafficking in health and disease: “PET metallomics”. RSC Chem. Biol..

[11] Turnlund J.R., Keyes W.R., Peiffer G.L. (1995). Molybdenum absorption, excretion, and retention studied with stable isotopes in young men at five intakes of dietary molybdenum. Am. J. Clin. Nutr..

[12] Hille R., Nishino T., Bittner F. (2011). Molybdenum enzymes in higher organisms. Coord. Chem. Rev..

[13] Lipiński W. (2016). Evaluation of Polish Soils Abundance in Microelements. Zesz. Nauk. UP Wroc..

[14] https://www.pgi.gov.pl/srodowiskowa/bloki-tematyczne/ates/baza-wiedzy/1309-warunki-hydrogeologiczne-a-funkcjonowanie-systemow-ates/15939-wartosci-stezen-zelaza-i-manganu-w-wynikach-oznaczen-chemicznych-z-lat-1991-2024-z-bazy-monitoring-wod-podziemnych-mwp-a-wytyczne-dla-systemow-ates-w-tym-zakresie.html.

[15] Bombik E., Bombik A., Pietrzkiewicz K. (2023). Content of Lead and Cadmium in the Tissues and Organs of the Wild Mallard Duck (*Anas platyrhynchos* L.) Depending on the Region of Poland Where It Is Harvested. Animals.

[16] Sejm (1995). Act of 13 October 1995—Hunting Law. J. Laws.

[17] Sejm (2015). Act of 15 January 2015 on the protection of animals used for scientific or educational purposes. J. Laws.

[18] StatSoft, Inc. (2015). Statistica (Data Analysis Software System), Version 13.0. https://statistica.software.informer.com/13.0/.

[19] Kalisińska E., Salicki W., Mysłek P., Kavetska K.M., Jackowski A. (2004). Using the mallard to biomonitor heavy metal contamination of wetlands in North-Western Poland. Sci. Total Environ..

[20] Rodríguez-Álvarez M., Paz S., Hardisson A., González-Weller D., Rubio C., Gutiérrez Á.J. (2022). Assessment of Toxic Metals (Al, Cd, Pb) and Trace Elements (B, Ba, Co, Cr, Cu, Fe, Mn, Mo, Li, Zn, Ni, Sr, V) in the Common Kestrel (*Falco tinnunculus*) from the Canary Islands (Spain). Biol. Trace Elem. Res..

[21] Horai S., Watanabe I., Takada H., Iwamizu Y., Hayashi T., Tanabe S., Kuno K. (2007). Trace element accumulations in 13 avians species collected from the Kanto area, Japan. Sci. Total. Environ..

[22] Kalisińska E., Salicki W., Jackowski A. (2006). Six trace metals in white-tailed eagle from Northwestern Poland. Pol. J. Environ. Stud..

[23] Bojar H., Bojar I. (2009). Monitoring of contamination of the Lublin region wetlands using mallards (*Anas platyrhynchos*) as a vector of the contamination by various conditionally toxic elements. Ann. Anim. Sci..

[24] Dżugan M., Zielińska S., Hęclik J., Pieniążek M., Szostek M. (2012). Evaluation of heavy metals environmental contamination based on their concentrations in tissues of wild pheasant (*Phasianus colchicus* L.). J. Microbiol. Biotechnol. Food Sci..

[25] Sujak A., Wiącek D., Jakubas D., Komosa A., Kitowski I. (2019). Mallards *Anas platyrhynchos* shot in Eastern Poland: Ecological risk evaluated by analysis of trace elements in liver. Hum. Ecol. Risk Assess. Int. J..

[26] Kim J., Oh J.-M. (2012). Metal levels in livers of waterfowl from Korea. Ecotoxicol. Environ. Safe.

[27] Majewska D., Szczerbińska D., Ligocki M., Bucław M., Sammel A., Tarasewicz Z., Romaniszyn K., Majewski J. (2016). Comparison of the mineral and fatty acid profiles of ostrich, turkey and broiler chicken livers. Br. Poult. Sci..

[28] Durkalec M.M., Nawrocka A., Kitowski I., Filipek A., Sell B., Kmiecik M., Jedziniak P. (2023). Lead, cadmium, and other trace elements in the liver of golden eagles and white-tailed eagles: Recent data from Poland and a systematic review of previous studies. Environ. Sci. Pollut. Res..

[29] Kim J., Oh J.-M. (2016). Assessment of trace element concentrations in birds of prey in Korea. Arch. Environ. Contam. Toxicol..

[30] Kalisińska E., Budis H. (2019). Manganese, Mn. Mammals and Birds as Bioindicators of Trace Element Contaminations in Terrestrial Environments.

[31] Al-Obaidi F.A., Mehdi B.I., Al- Shadeedi S.M. (2013). Identification of some inorganic elements in tissues of some wild birds in Iraq. J. Genet. Environ. Res. Conserv..

